# Electrostatic pollination by butterflies and moths

**DOI:** 10.1098/rsif.2024.0156

**Published:** 2024-07-24

**Authors:** Sam J. England, Daniel Robert

**Affiliations:** ^1^ School of Biological Sciences, Faculty of Life Sciences, University of Bristol, Bristol, UK

**Keywords:** electric fields, flowers, insects, Lepidoptera, pollen, static charge

## Abstract

Animals, most notably insects, generally seem to accumulate electrostatic charge in nature. These electrostatic charges will exert forces on other charges in these animals’ environments and therefore have the potential to attract or repel other objects, for example, pollen from flowers. Here, we show that butterflies and moths (Lepidoptera) accumulate electrostatic charge while in flight. Then, using finite element analysis, we demonstrate that when within millimetres of a flower, the electrostatic charge of a lepidopteran generates an electric field in excess of 5 kV m^−1^, and that an electric field of this magnitude is sufficient to elicit contactless pollen transfer from flowers across air gaps onto the body of a butterfly or moth. Furthermore, we see that phylogenetic variations exist in the magnitude and polarity of net charge between different species and families and Lepidoptera. These phylogenetic variations in electrostatic charging correlate with morphological, biogeographical and ecological differences between different clades. Such correlations with biogeographical and ecological differences may reflect evolutionary adaptations towards maximizing or minimizing charge accumulation, in relation to pollination, predation and parasitism, and thus we introduce the idea that electrostatic charging may be a trait upon which evolution can act.

## 1. Introduction

Many species of airborne animal have been shown to naturally accumulate electrostatic charges as they fly through their environment [1–10]. Because electrostatic charges generate electric fields that exert forces on other charged objects, they have the potential to be mechanistically involved in the ecological interactions of these charge-carrying animals. For example, it has been suggested that the electrostatic charges of animals could be implicated in their susceptibility to predation and parasitism, via electrostatic attraction of spider webs [11,12], ticks [13] and mites [14], as well as increasing their detectability to electroreceptive predators [10]. Most pertinently here, it has been hypothesized that electrostatic forces may play an important role in zoophilous pollination [1,8,15–17]. This is because most animals tend to accumulate positive charges [1,17], while pollen grains regularly carry negative charges [18,19]. Therefore, there will be an attractive force between pollen and animal because opposite charges attract each other. Ultimately, this can lead to an increase in the pollination efficiency of pollen vectors when they carry electrostatic charge and even allow for contactless pollen transfer from plant to pollinator. Indeed, theoretical and inferential evidence strongly suggests that the charges naturally accumulated by honeybees [15], bumblebees [17] and a species of hummingbird [8] are of sufficient magnitude to attract pollen onto their surfaces. This pollen can then be deposited on subsequently visited flowers, either by direct contact, or similarly through electrostatic attraction, because the pollen can equalize to the potential of the pollinator, and will then experience an attractive force towards the electric field of the flower [16,17,20,21]. Experimental evidence demonstrates this bidirectional electrostatic pollen transfer [17]. However, it is not clear whether these hypotheses and calculations are applicable to the wider variety of equally important pollinator taxa found in nature. In order to address this, we investigated the electrostatics of pollination in butterflies and moths (Lepidoptera).

We chose to investigate the role of electrostatics in pollination by Lepidoptera for several reasons. First, the Lepidoptera are seen to be key and widespread pollinators [22–24], with some species even having developed obligate mutualisms with specific plant species [25–27]. This ubiquity and co-evolutionary history have therefore made them vital for healthy ecosystem functioning in many parts of the world. Understanding the physical mechanisms of pollination by lepidopterans will be key to elucidating the evolutionary drivers acting on them and the angiosperms that they pollinate.

Furthermore, in the case of butterflies, whether they act as pollinators has been deemed controversial by some authors. A few ecological studies suggest that butterflies are poor pollinators, and thus act more akin to parasites of the flowers they visit, robbing nectar without providing pollination services in return [28,29]. This supposed pollination inefficiency may be supported if it is found that some butterfly species do not carry sufficient electrostatic charges to attract pollen. Conversely, if butterflies are found to carry electrostatic charges comparable to other proven pollinators such as bees and hummingbirds, it is more likely that butterflies contribute to pollination. Identifying electrostatic charging as a trait supporting pollen transfer would help in the documentation of pollination relationships in ecological networks.

Additionally, no quantitative measures of electrostatic charges carried by butterflies and moths currently exist. It cannot be assumed that their charges will be comparable to other animals previously measured because it is thought that flying animals charge via friction with the air as they flap their wings [1,17]. The Lepidoptera are notable for their wingbeat frequencies generally being up to two orders of magnitude lower than most other pollinators [30–32]; however, their wings constitute a much higher proportion of their body surface area than most other pollinators. How each of these factors interact to influence the electrostatic charging of butterflies and moths is not known.

Finally, the Lepidoptera exhibit a surprising diversity in their ecological functions and niches. While some species visit flowers, many do not feed at all as adults and therefore will almost never act as pollinators. In addition, different lepidopteran species exhibit diurnal, nocturnal and crepuscular activity regimes, are found in a variety of climates and habitats and employ various strategies of feeding at flowers, i.e. landing versus hovering. A very small number of lepidopteran species have even evolved to engage in pollinivory [33–35], meaning that they will stand to benefit directly from gathering pollen, unlike their non-pollinivorous relatives. By investigating lepidopteran taxa across this range of ecological, evolutionary and biogeographical contexts, a comparative approach can be employed to identify any correlations between these factors and the typical electrostatic charges that each butterfly and moth carries.

In this study, we hypothesize that butterflies and moths will generally carry an electrostatic charge of sufficient magnitude that it will have a tangible positive effect on their efficiency as pollinators. We also hypothesize that variations in the average electrostatic charge exist between species, and that these variations correlate with morphological differences between the species, such as size, but also ecological, biogeographical and life history traits. This hypothesis posits that the affinity of an organism to accumulate or maintain charge is a heritable characteristic, for example, via modification of the microstructure or chemical composition of its outer surfaces, and that the tendency to possess certain charge magnitudes or polarities may be adaptive in different environments. Thus, chargeability may be naturally selected for in certain evolutionary lineages and scenarios.

**Figure 1.**
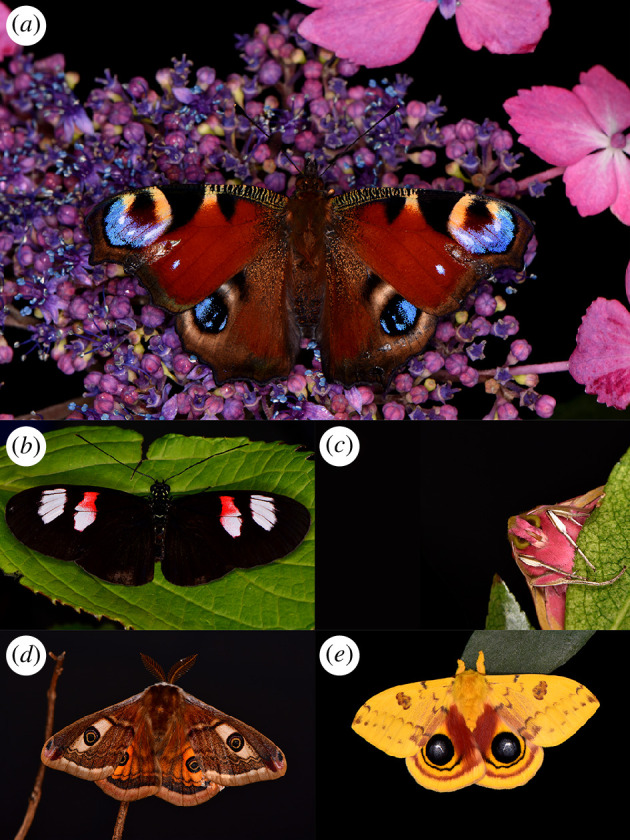
Macrophotographs of 5 of 11 lepidopteran species investigated within this study. (*a*) European peacock butterfly (*Aglais io*). (*b*) Postman butterfly (*Heliconius melpomene*). (*c*) Elephant hawk moth (*Deilephila elpenor*). (*d*) Male Ligurian emperor moth (*Saturnia pavoniella*). (*e*) Male Io moth (*Automeris io*).

Herein, in pursuit of testing these hypotheses, we provide the first quantitative measurements of the net electrostatic charge carried by lepidopteran taxa, surveying 11 different species (figure 1) from five different families, native to five different continents, and that employ a broad range of ecological strategies. These data are then input into finite element analysis models in order to computationally assess the likelihood of electrostatic forces playing a role in pollination by lepidopterans.

## 2. Material and methods

### 2.1. Sourcing and care of animals

All animals were housed in mesh enclosures exposed to climatic conditions comparable to their natural environments. All species obtained as eggs or caterpillars were fed appropriate foodplants ad libitum until pupation. All species that feed as adults were provided with a sugar source, in the form of sugar solution (in artificial flowers or hand-fed by pipette) or pineapple slices. Species-specific details on their sourcing and care are provided in electronic supplementary material, table S1.

### 2.2. Charge measurements

The net charges of the butterflies and moths were measured using a modified version of a methodology previously published [7,10]. This method uses a picoammeter connected to a shielded ring electrode system that produces voltage curves (figure 3*a*) with integrals proportional to the magnitude, and directions based on the polarity, of any charge that passes through the rings. This system is described in detail in the electronic supplementary material.

To measure the charge of free-flying butterflies, adult peacock butterflies (*Aglais io*; *n* = 72) had their net charge recorded as they flew from the exit of a container lined with leaves, through the ring electrode system. Leaves were chosen to line the container so that any charge the butterflies accumulated would either be through flight or contact with a surface that they would encounter in their natural habitat, ensuring the charge measurements are ecologically relevant. Then, to understand how electrostatic charge might vary across species, 10 further species of butterfly and moth had their net electrostatic charge measured, namely the small tortoiseshell (*Aglais urticae; n* = 34), the cinnabar moth (*Tyria jacobaeae*; *n* = 8), the elephant hawkmoth (*Deilephila elpenor*; *n* = 17), the postman butterfly (*Heliconius melpomene*; *n* = 28), the Julia butterfly (*Dryas iulia*; *n* = 27), the eastern tiger swallowtail (*Papilio glaucus*; *n* = 3), the Ligurian emperor moth (*Saturnia pavoniella*; *n* = 20), the spongy moth (*Lymantria dispar*; *n* = 29), the Suraka silk moth (*Antherina suraka*; *n* = 5) and the Io moth (*Automeris io*; *n* = 26). These species had their charges measured by dropping tethered individuals through the ring electrode system after a minimum of 30 s of flight. See the electronic supplementary material for more details on charge measurement protocols.

### 2.3. Modelling of electric field strengths and pollen trajectories

The electric field strength arising between a charged lepidopteran and a flower, as well as the movement of pollen that this would elicit, was modelled using three-dimensional finite element analysis in COMSOL Multiphysics® v. 5.4 (COMSOL AB, Stockholm, Sweden). The surface of the butterfly representation was assigned a charge of +54.5 pC, derived from the mean magnitude of charge measured for free-flying *Aglais io* in this study (*n* = 72; figure 3*b*). For simplicity, the flower has a singular anther at its centre. Each pollen grain was subject to three forces: an electric force derived from the pollen grain’s charge and the electric potential at the pollen grain’s location, a drag force assumed to follow Stokes’ law, and Earth’s gravity. See electronic supplementary material for model geometry and parameter details.

### 2.4. Statistics

All statistical analyses were performed in R 4.2.1 [36]. Because the magnitude of the net charge held by a lepidopteran will dictate the magnitude of the force on other charged objects in their environment, while the polarity of the charge will dictate the direction of said force, these two qualities of the net charge probably have distinct biophysical and ecological implications. Therefore, the magnitude and polarity of the net charge were tested for statistical variations separately. The charge magnitude data were transformed before analysis with a log(*x* + 1) transformation, ensuring that the data fitted a normal distribution while not producing any undefined or negative values, and then examined with linear mixed-effects models (LMM), whereas the charge polarity data were examined with generalized linear mixed-effects models (GLMM) using a binomial distribution specification. In all models, the day that the charge measurement was made was included as a random effect, in order to control for variations in environmental factors such as humidity, temperature and ion concentration that occurred between days in the laboratory. The variables of interest included phylogenetic family, species and surface area, *A*, approximated to be that of a prolate spheroid body with infinitesimally flat wings attached to it, calculated as


$$A=2\left[S_{All}+\;\pi r_{L}r_{W}\left(\left(\frac{r_{W}}{r_{L}}+\frac{\alpha }{\sin\left(\alpha \right)}\right)-1\;\right)\right],$$

where α is the angular eccentricity of the body,


$$\alpha =\cos^{-1}\left(\frac{r_{W}}{r_{L}}\right).$$

**Figure 2.**
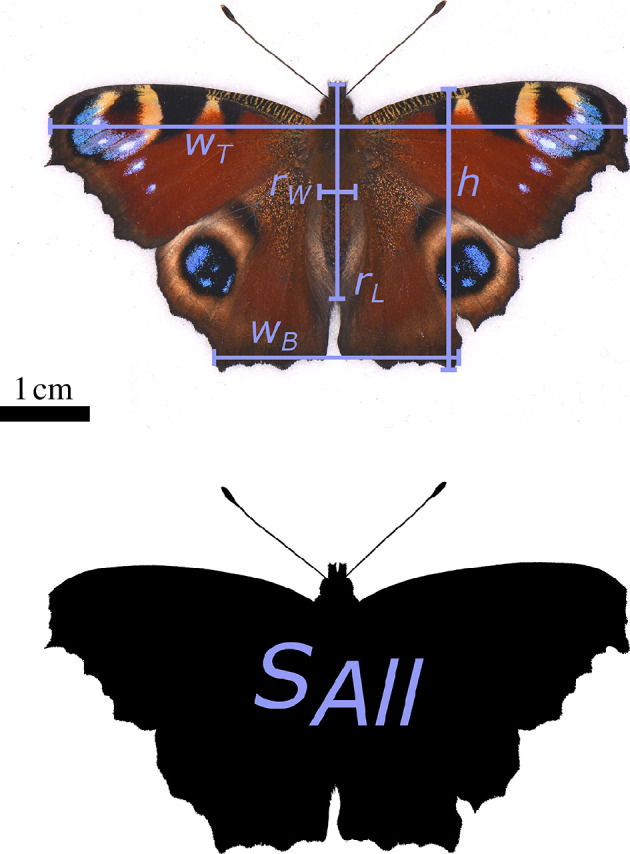
Example measurements of the variables *r*_L_, *r*_W_ and *S*_All_, as introduced and required for the equation derived for the surface area of a lepidopteran, as well as *w*_T_, *w*_B_ and *h*, used for building a scale interpretation of a butterfly for finite element analysis. This example is applied to a peacock butterfly (*Aglais io*).

Figure 2 and the electronic supplementary material explain variable definitions and measurement, along with formula derivation. Ecological variables, namely floral visitation, activity regime (together, ‘ecological guild’) and native climate, were also included. The effect of each variable was determined by conducting multiple likelihood ratio tests against a Chi-squared distribution, with subsequent pairwise post hoc tests for some groups. For further details on the statistical approach, see electronic supplementary material.

## 3. Results

### 3.1. The electrostatic charge of free-flying butterflies

**Figure 3.**
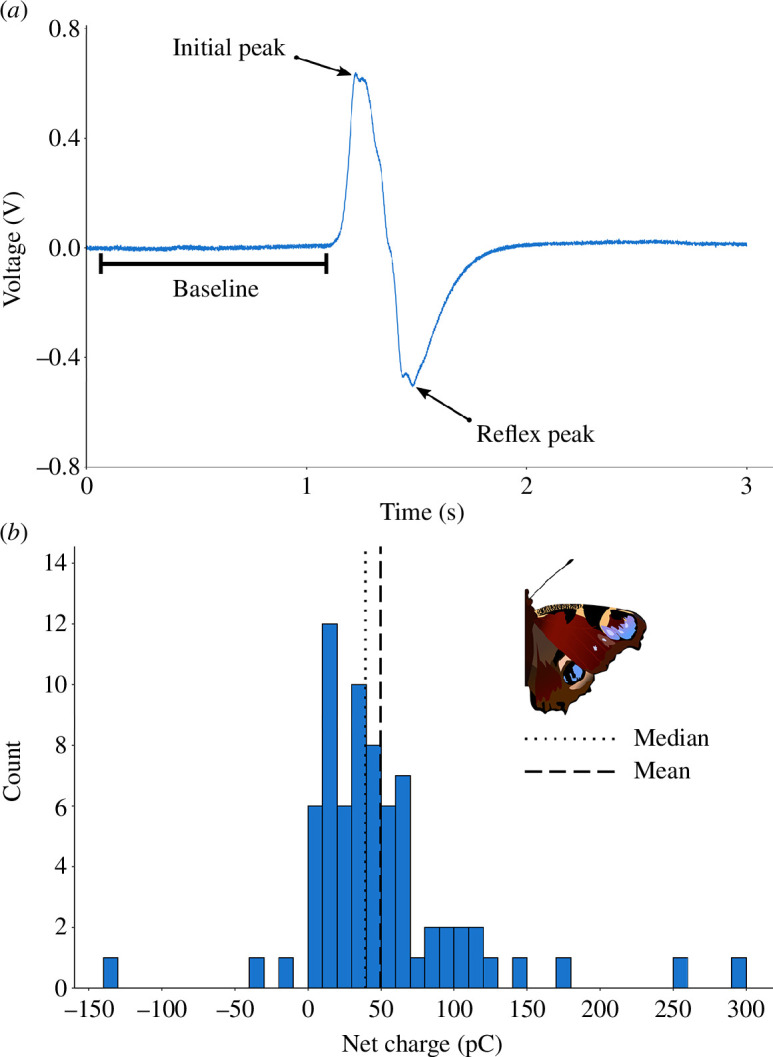
(*a*) A typical example of the voltage generated by the picoammeter over time as a charged lepidopteran flew freely through the ring electrode system. An offset has been applied to bring the baseline to 0, and a notch filter between 49 and 51 Hz to reduce 50 Hz noise from mains electricity in the United Kingdom. This particular example was a peacock butterfly (*Aglais io*) carrying a net electrostatic charge of +15.41 pC. (*b*) Distribution of net electrostatic charges carried by free-flying *Aglais io* butterflies. Mean ± s.d. = +49.54 ± 57.22 pC, median = 39.40 pC, *n* = 72.

All free-flying *Aglais io* individuals measured carried a non-zero net electrostatic charge (*n* = 72; figure 3*b*). The average net charge was +49.54 ± 57.22 pC (mean ± s.d.). The net charge ranged from a minimum of −132.68 pC to a maximum of 299.93 pC, with a median of 39.40 pC. The mean magnitude of the net charge was 54.53 pC. The net charges were almost always positive in polarity, with 96% (*n* = 69) carrying a net positive charge, and 4% (*n* = 3) carrying a net negative charge.

### 3.2. Computational modelling of electrostatic pollen transfer

**Figure 4.**
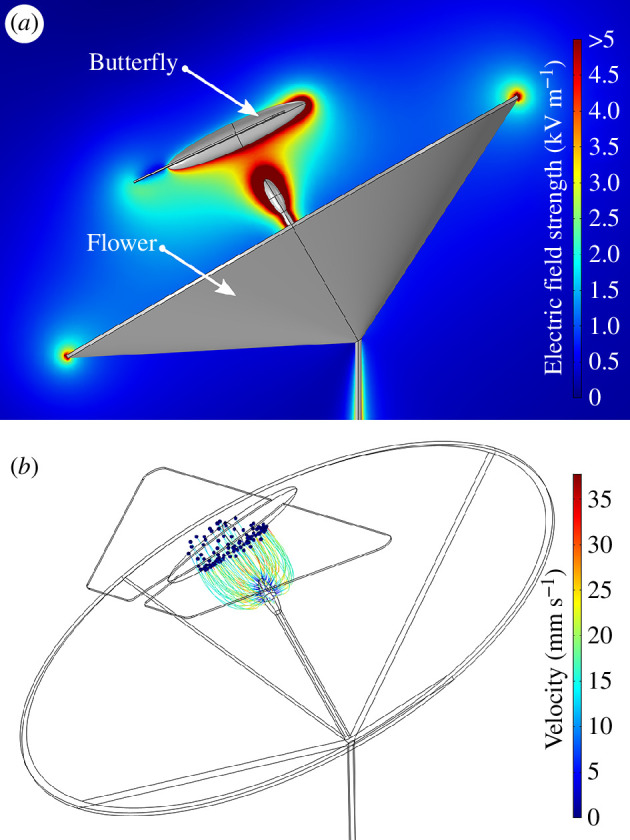
(*a*) Three-dimensional computational model of the electric field formed between a typically charged lepidopteran and a grounded flower. Colour scale represents electric field strength, with data truncated above 5 kV m^−1^ for clarity. Grey indicates model geometry. (*b*) Three-dimensional computational model simulating pollen grain trajectories (*n* = 100) under the influence of electric, gravitational and drag forces, for a typically charged lepidopteran positioned 6 mm from the stamen of a flower. Blue circles show the final locations of individual pollen grains. Colour scale represents the velocity of pollen grains. All models are produced using the finite element method.

The computational model of the electric field generated between a typically charged butterfly and a grounded flower shows that electric fields of considerable magnitude exist during floral visitations by lepidopterans, upwards of 5 kV m^−1^ (figure 4*a*). This electric field is strongest around the anther of the flower. Simulation of the trajectories of pollen grains under the influence of this electric field, as well as gravity and drag forces, shows that the average charge magnitude of a butterfly creates sufficient electrostatic force to lift pollen grains across an air gap of at least 6 mm, with all 100 pollen grains in the simulation contacting the butterfly within 1 s (figure 4*b*).

### 3.3. Phylogenetic, ecological and biogeographical variations

**Figure 5.**
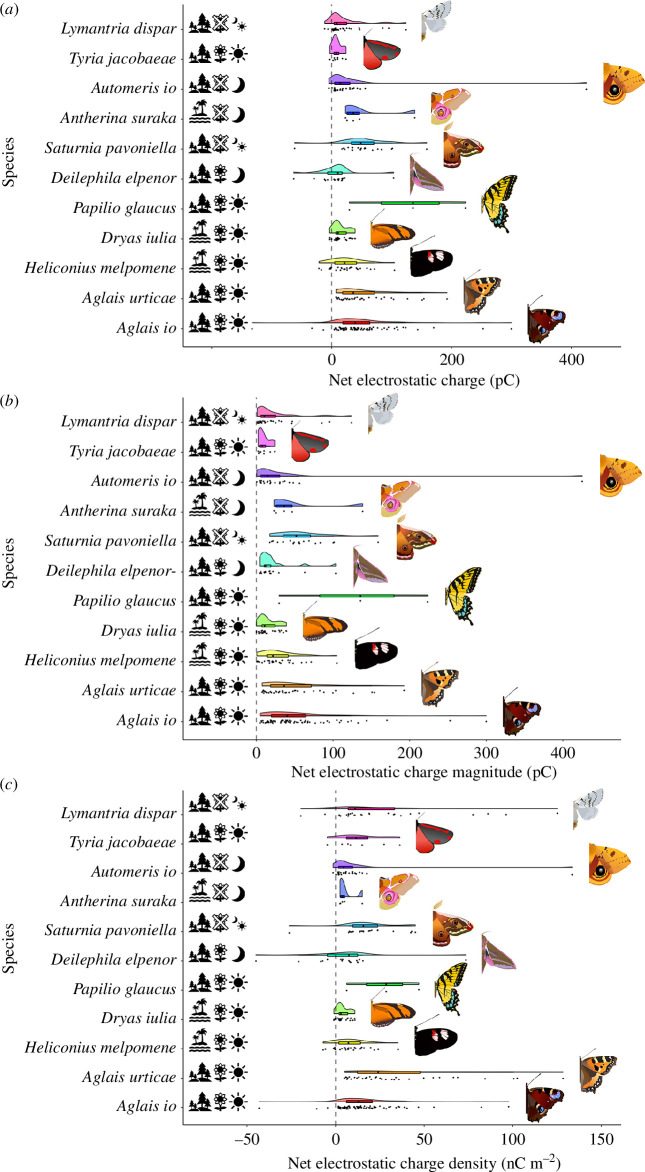
Different characteristics of the net electrostatic charge carried by various species of butterfly and moth. Conifer trees denote temperate species, palm trees denote tropical species, flowers denote floral visitors, flowers crossed-through denote species that do not visit flowers, suns denote diurnal species and moons denote nocturnal species. The presence of both a sun and moon denotes that intraspecies differences in the activity regime exist between sexes, with the females’ activity regime indicated by the top left icon, and the males’ activity regime indicated by the bottom right icon. Points indicate individual measurements; box and whisker show the median, lower and upper quartiles and range; half-violin shows the distribution of each dataset. (*a*) The net electrostatic charge. (*b*) The net electrostatic charge magnitude. (*c*) The net electrostatic charge density is calculated by normalizing the net electrostatic charge by an estimation of each species’ surface area.

Across all 11 species tested, every individual carried a non-zero net electrostatic charge, with variations in the charge distributions visible between species (figure 5*a*). Interspecies variability can also be seen in the charge magnitude (figure 5*b*), charge density (figure 5*c*) and the relative frequency of each charge polarity. For charge magnitude, the LMM with phylogenetic family as a fixed effect, and day of measurement as a random effect was a significantly better fit than the LMM without family as a fixed effect (χ^2^ = 22.949, d.f. = 4, *p* = 0.0001296). Therefore, the magnitude of charge held by a lepidopteran varies significantly at the family level. The LMM with species as a fixed effect, and family and day of measurement as random effects was a significantly better fit than the LMM without species as a fixed effect (χ^2^ = 31.472, d.f. = 10, *p* = 0.0004901). Therefore, the magnitude of charge held by a lepidopteran varies significantly at the species level, independent of any family-level variations. A pairwise comparison performed on this model between the pollinivorous *Heliconius melpomene*, and a non-pollinivorous close-relative, *Dryas iulia* yielded no significant difference in the model predictions of charge magnitude between these species (*z* = 0.528, *p* = 0.598).

**Figure 6.**
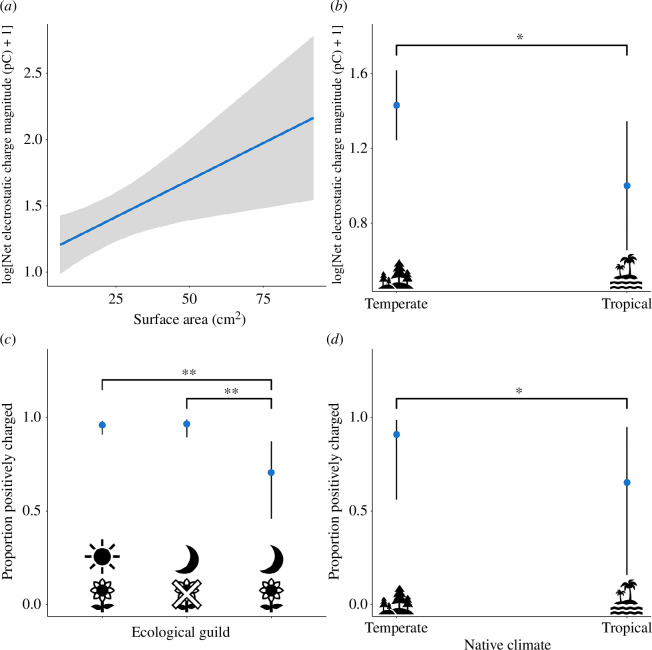
Predicted values based upon surface area (*a*) and native climate (*b*), from the linear mixed-effects model for the transformed net electrostatic charge magnitude, with surface area and native climate as fixed effects, and day of measurement, species and family as random effects. Predicted values based upon ecological guild (*c*) and native climate (*d*), from the generalized linear mixed-effects model for the net electrostatic charge polarity, with ecological guild and native climate as fixed effects, and day of measurement and species as random effects. Blue denotes predicted values, lines or the shaded area denote 95% confidence intervals. Asterisks indicate statistically significant differences: * indicates *p* < 0.05, ** indicates *p* < 0.01.

Because *a priori* hypotheses could be conceived for potential interactions between the native climate, floral visitation and activity regime of the lepidopterans, two LMMs for the charge magnitude were constructed with these factors, alongside surface area, as fixed effects, and day of measurement, species and family, as random effects; one model contained interaction terms between native climate, floral visitation and activity regime, and the other did not. No significant difference was found between these two models (*χ* ^2^ = 3.1154, d.f. = 2, *p* = 0.2106), and therefore it was deemed unnecessary to include interaction terms in subsequent models. Sequentially removing one of the fixed effects from the LMM with surface area, native climate, floral visitation and activity regime as fixed effects, and day of measurement, species and family as random effects, and comparing to the full model with the use of ANOVAs, showed that the surface area (*χ* ^2^ = 7.8398, d.f. = 1, *p* = 0.005111) and native climate (*χ* ^2^ = 5.4321, d.f. = 1, *p* = 0.01977) of the lepidopteran are significant predictors of the charge magnitude, whereas floral visitation (*χ* ^2^ = 1.6653, d.f. = 1, *p* = 0.1969) and activity regime (*χ* ^2^ = 0.0441, d.f. = 1, *p* = 0.8337) are not. Repeating this process to the same LMM but with floral visitation and activity regime removed maintained surface area (*χ* ^2^ = 7.1202, d.f. = 1, *p* = 0.007622) and native climate (*χ* ^2^ = 4.2863, d.f. = 1, *p* = 0.03842) as significant explanatory factors for the net charge magnitude of lepidopterans. Plotting the predicted values from this model shows that a higher surface area predicts a higher net charge magnitude (figure 6*a*), and that tropical species are predicted to have a lower net charge magnitude than temperate species, irrespective of the climatic conditions within which they are measured (figure 6*b*).

For the net charge polarity, the GLMM with family as a fixed effect and day of measurement as a random effect was not a significantly better fit than the GLMM without family as a fixed effect (*χ* ^2^ = 8.2536, d.f. = 4, *p* = 0.08272). Therefore, phylogenetic family is not a significant predictor of the net charge polarity of lepidopterans and was not controlled for in subsequent models. However, a pairwise comparison between the model predictions for hawkmoths and Sphingidae, was significantly different to their closest relative the saturniids, Saturnidae (*z* = −2.547, *p* = 0.0109). As such, hawkmoths are more likely to carry a negative charge than saturniids. The GLMM with species as a fixed effect and day of measurement as a random effect was a significantly better fit than a GLMM without species as a fixed effect (*χ* ^2^ = 20.67, d.f. = 10, *p* = 0.02351). Therefore, species is a significant predictor of the charge polarity of lepidopterans. The GLMMs with native climate, activity regime and floral visitation as fixed effects with interaction terms, and day of measurement and species as random effects, was a significantly better fit than the same model without the fixed effects having interaction terms (*χ* ^2^ = 8.2712, d.f. = 2, *p* = 0.01599). To deal with this, activity regime and floral visitation were combined into a single ‘ecological guild’ variable with four groups: diurnal floral visitors, nocturnal floral visitors, diurnal non-visitors and nocturnal non-visitors. The two GLMMs with native climate and ecological guild as fixed effects (with interaction terms in one model and without in the other) and day of measurement and species as random effects, did not show a significant difference in the quality of fit (*χ* ^2^ = 2.2289, d.f. = 1, *p* = 0.1354). Therefore, interaction terms were not included in the final models. Comparing the fit of this GLMM with GLMMs with one of the fixed effects sequentially removed, showed that both the native climate (*χ* ^2^ = 5.8328, d.f. = 1, *p* = 0.01573) and ecological guild (*χ* ^2^ = 9.8621, d.f. = 3, *p* = 0.01978) of the lepidopterans are significant predictors of the charge polarity. Plotting the model predictions shows that tropical species are more likely to be negatively charged than temperate species (figure 6*d*). Furthermore, pairwise comparisons reveal that nocturnal floral visitors are more likely to be negatively charged than their diurnal floral visitor (*z* = −3.286, *p* = 0.00196) and nocturnal non-visitor (*z* = −3.033, *p* = 0.00463) counterparts, suggesting that floral visitation and activity regime may interact to influence the charge polarity of lepidopterans (figure 6*c*).

## 4. Discussion

Overall, this study demonstrates clearly that butterflies and moths accumulate a net electrostatic charge. All individuals measured, from various phylogenetic, ecological and biogeographical groupings, carried a net electrostatic charge, suggesting that electrostatic charging is a universal trait among the Lepidoptera. The magnitudes of these net electrostatic charges are commensurate with those measured on other animals and are generally higher than the charges measured on other insects [1]. This shows that despite their wingbeat frequency being about two orders of magnitude lower than most other insects [30–32], butterflies and moths are still capable of accumulating appreciable electrostatic charge.

Importantly, the magnitude of the net electrostatic charge on the lepidopterans measured is sufficient to facilitate contactless pollination by species that visit flowers, via electrostatic attraction of pollen grains across air gaps of several millimetres. This finding supports the hypothesis that butterflies indeed act as pollinators, despite some suggestions otherwise [28,29].

Promisingly, our measurements and models are probably underestimating the strength and influence of electrostatic forces in zoophilous pollination for several reasons. First, it has previously been shown that the net electrostatic charge of bees is typically higher when measured in the field, as compared with laboratory measurements [7]. Therefore, the charge values for lepidopterans measured in this study may have underestimated the net charge that butterflies and moths accumulate in nature. Furthermore, our measurements of charge can only inform us of the net charge carried by each individual. It is likely that different body regions of each lepidopteran may be charged to opposite polarities, which would be cancelled out for any measure of the net charge. As such, the total amount of charge carried by each lepidopteran is probably higher than the net charge, and at close range, this higher total charge will provide even greater forces than estimated by the net charge. Even further, the measurements of net electrostatic charge will not be able to detect electrical polarizations of the lepidopteran tissues, which would have an apparent electric field that could attract pollen at close range while having a net zero charge across the whole animal. Indeed, the wings of butterflies have previously been shown to be piezoelectric, meaning electric polarizations can be generated within them by applied mechanical stresses [37,38], which could occur as the wings are contorted in flight. Our models also do not take into account the electrical polarization of pollen grains which would similarly make them more strongly attracted to a charged lepidopteran. Finally, our models do not consider the aerodynamics of lepidopteran flight. The airflow induced by wing flaps probably disturbs pollen grains and thrusts them into the air, potentially making them more available for subsequent electrostatic capture. Furthermore, the lift mechanisms used by butterflies and moths during flight necessitate the generation of vortices across the wings’ surfaces [39–47]. It is possible that these vortices trap pollen grains and repeatedly cycle them in close proximity to the wings, further increasing their vulnerability to electrostatic capture. Future studies on zoophilous electrostatic pollination should consider these potentially synergistic effects of flight fluid dynamics.

The data herein also clearly demonstrate that phylogenetic variations exist in the charge magnitude and polarity at the interspecies and interfamily levels. The primary driver of this interspecies variation was seen to be the surface area of the lepidopteran, which is an intuitive finding because a larger surface area permits the accumulation of a greater charge without increasing charge density (which would require more energy to maintain). This is the first evidence of an allometry existing in the scaling of the electrostatic charges held by animals.

However, variations in electrostatic charge were also correlated with biogeographical and ecological factors. We hypothesize that these interspecies variations may therefore be evolutionarily adaptive. Possessing a high electrostatic charge may be naturally selected for owing to a few reasons. First, in the case of flower-visiting species, it is possible that a more efficient pollinator would be selected for, because it will result in more flowers of that species being available as a resource for the lepidopteran’s descendants. This effect is probably marginal, and vulnerable to cheaters, but will be especially strong among lepidopterans that have formed more exclusive mutualisms with specific plant species. Furthermore, other pollinating insects have previously been shown to be capable of detecting the electric fields around flowers and may use this information as a sensory cue to identify flowers that have recently been visited, thus avoiding a diminished nectar reward [6,48]. Importantly, it has been shown that the higher the charge carried by these insects, the higher the sensitivity of their electroreception [49–52]. Therefore, if butterflies or moths also use electroreception to assess potential floral rewards, carrying a higher net charge would improve their foraging efficiency. Indeed, the likelihood of butterflies and moths being capable of electroreception is bolstered by the recent discovery that their caterpillars, including two of the species examined in this study, are electroreceptive [10]. Even further, the electrostatic charge of visiting insects has been implicated in the triggering of physiological processes in flowering plants, and may even act as a signal to the plant [53]. It is conceivable that these physiological responses to insect charge may include increased nectar production. Therefore, carrying a higher net charge would benefit flower-visiting lepidopterans by improving the quality of reward available at each flower. However, there are also reasons that carrying a high net charge would be maladaptive for lepidopterans. First, the accumulation of pollen on insects hinders their flight ability, with detrimental effects on manoeuvrability and metabolic output [54,55]. In addition, possessing a high electrostatic charge may make lepidopterans more vulnerable to predation and parasitism. It has previously been shown that spider webs are electrostatically attracted towards positively charged insects [11], and suggestions have been made that ectoparasites may similarly be attracted by the charge of their insect hosts [14,56]. Beyond this, because it appears that aerial electroreception may be a widespread sense among terrestrial animals [1,6,10,48,57,58], it is conceivable that animals predating upon lepidopterans, particularly floral ambush predators, may use the static charge of their prey as a sensory cue. In fact, it has already been shown that prey animals can detect their predators via electroreception in air [10], and thus electroreceptive detection of prey by predators is highly likely. Therefore, possessing a low net charge, or a negative charge atypical for animals [1], may preclude detection by predators. Indeed, altogether, it is likely that a series of interconnected and competing selective pressures may be applied to the electrostatic charging of lepidopterans; the relative weighting and importance of which will vary with ecological and biogeographical factors.

It is interesting that the pollinivorous *Heliconius melpomene* did not carry a significantly different charge magnitude to its non-pollinivorous close relative *Dryas iulia*, because a clear adaptive benefit for electrostatic charging can be hypothesized for *Heliconius*, in that it has the potential to increase their pollen-gathering efficiency. However, the lack of any such adaptation can easily be explained by the fact that *Heliconius* specifically gather pollen on their proboscis, and are unlikely to be able to retrieve pollen adhered to other parts of their bodies. Therefore, the net charge of the entire body of the butterfly is an irrelevant measure in this case. Future studies should aim to obtain a quantitative measure of the charge specifically on the proboscises of *Heliconius* and its non-pollinivorous relatives, which may reveal more marked differences. Indeed, morphological and behavioural variations related to the *Heliconius* proboscis, such as a higher density of bristles and more frequent grooming [34,35,59–61], are likely to increase its charge compared with the proboscises of other butterflies.

Fascinatingly, the finding that tropical lepidopterans carry lower net charge magnitudes, and are more likely to be negatively charged, may inform us about contrasts between the electric ecology of tropical and temperate environments. One possible explanation for this discrepancy is that, in higher humidity, the magnitude of surface charge accumulated by animals appears to be lower [7,8]. Because humidity is controlled for in our statistical models, this may suggest that less evolutionary pressure has been applied to adaptive charge accumulation traits within tropical species because the physical environment of the tropics limits the extent to which any charge can be accumulated. Alternatively, the discrepancy in charge between tropical and temperate species may reflect adaptions for reducing charge accumulation, because predation pressure on insects is known to be higher in the tropics [62]. Similarly, the finding that nocturnal floral visitors are more often negatively charged than diurnal floral visitor and nocturnal non-visitor species, may also be a product of increased predation risk. This is because nocturnal floral visitation is inherently risky, as many ambush predators tactically locate themselves on flowers and will be harder to avoid visually at night [63–75]. Furthermore, owing to the diminished reliability of visual cues at night, nocturnal predators are more likely to rely on other sensory modalities, such as electroreception, for prey detection.

Altogether, this study demonstrates that the electrostatic charging of butterflies and moths impacts their ecology, and as such, potentially their evolution too. We have unveiled a complicated set of interacting and competing factors in determining the magnitude and polarity of lepidopteran charge that warrant further investigation to fully disentangle the mechanisms, drivers and consequences of interspecies diversity in electrostatic charging.

## Data Availability

All data have been deposited in Mendeley Data and are publicly available as of the date of publication [76]. Supplementary material is available online [77].
